# EFFECT OF CALORIC RESTRICTION AND RAPAMYCIN ON OVARIAN AGING IN MICE

**DOI:** 10.1093/geroni/igz038.385

**Published:** 2019-11-08

**Authors:** Driele Garcia, Tatiana Saccon, Joao Rincon, Jorgea Pradiee, Rafael Mondadori, Michal Masternak, Andrzej Bartke, Augusto Schneider

**Affiliations:** 1 Universidade Federal de Pelotas, Pelotas, RS, Brazil; 2 University of Central Florida, Orlando, Florida, United States; 3 Southern Illinois University School of Medicine, Springfield, Illinois, United States

## Abstract

The ovarian follicular reserve of primordial follicle declines with aging in female mammals. Caloric restriction (CR) has been shown to increase the preservation of the ovarian follicular reserve. Likewise, rapamycin has similar effects to CR on the ovarian reserve. Therefore, the aim of our study was to evaluate the effects of rapamycin and CR on the metabolism and ovarian follicular reserve and gene expression in mice. Thirty-six female mice were used, and allocated into 3 groups: control, rapamycin (4mg/kg body weight every other day) and 30% CR. At 85 days of treatment, an insulin tolerance test (ITT) and glucose tolerance test (GTT) was performed. At 93 days ovaries were collected for analysis. CR females had lower body weight (P<0.05) and were more insulin sensitive (P=0.003), while rapamycin treated females did not change body weight (P>0.05) and were more resistant to insulin (P<0.05). Females from the CR and rapamycin groups had a twice higher number of primordial follicles (P=0.02 and 0.04) and half the number of primary, secondary and tertiary follicles (P<0.05). Both CR and rapamycin females had increased ovarian gene expression of Foxo3a mRNA (P<0.05). In conclusion, female mice from rapamycin and CR groups had an increased ovarian follicular reserve associated to higher expression of Foxo3a mRNA, despite divergent metabolic effects of the treatments.

